# Patterns of multidrug resistance in *Salmonella* isolated from commerciallayers during 4 months of rearing in Mymensingh, Bangladesh

**DOI:** 10.5455/javar.2025.l975

**Published:** 2025-12-25

**Authors:** Anny Khatun, Md. Hadiuzzaman, Ashik Ahmed Durber, Sourav Chakraborty, Most. Nahida Khatun, Rupaida Akter Shila, Sakib Anzum Pranto, Ripon Sarker, Marzia Rahman, Mohammod Kamruj Jaman Bhuiyan, Md. Alimul Islam, Dipongkor Saha, Kazi Rafiq, Muhammad Tofazzal Hossain

**Affiliations:** 1 Department of Microbiology and Hygiene, Bangladesh Agricultural University, Mymensingh, Bangladesh; 2 Department of Pathology, Bangladesh Agricultural University, Mymensingh, Bangladesh; 3 Department of Agricultural and Applied Statistics, Bangladesh Agricultural University, Mymensingh, Bangladesh; 4 Department of Biology, North Carolina Agricultural and Technical State University, Greensboro, NC, USA; 5 Department of Pharmacology, Bangladesh Agricultural University, Mymensingh, Bangladesh

**Keywords:** AMR, Antibiotic resistance, Commercial layers, *Salmonella*

## Abstract

**Objective:**

Antibiotic-resistant bacterial pathogens from livestock and poultry pose a significant global concern, contributing to many foodborne and zoonotic diseases. This study aimed to detect *Salmonella* spp. from selected poultry farms during a defined study period, with a particular focus on antibiotic resistance.

**Materials and Methods:**

One hundred and five cloacal swabs were obtained aseptically from birds of seven randomly selected commercial layer farms of Mymensingh district in Bangladesh. The isolation of *Salmonella* spp. was performed through culturing on selective agar media and subsequently confirmed by polymerase chain reaction (PCR) using specific primers. The disc diffusion method was performed to determine the sensitivity of confirmed *Salmonella* spp. isolates against 19 antibiotics. Finally, PCR was performed to detect the tetracycline (*tet*A) and beta-lactamase (*bla*_TEM_) genes.

**Results:**

Out of 105 samples, 34 were detected as positive for *Salmonella* spp. on *Salmonella*-Shigella media, of which 20 (19.05%) isolates were confirmed as *Salmonella* spp. (211 bp). Erythromycin, cefuroxime, doxycycline, amoxicillin, ampicillin, and *tet*A were ineffective against all 20 isolates. Several unique antibiotic resistance patterns were observed, with most isolates exhibiting multidrug resistance (MDR). Furthermore, 100% of the phenotypically resistant isolates contained the *tet*A and *bla*_TEM_ genes.

**Conclusion:**

Commercial layers in Bangladesh were found to harbor MDR *Salmonella* spp., representing a potential risk to the poultry population and a public health concern.

## Introduction

Poultry farming has become a profitable venture in Bangladesh, contributing to employment opportunities and national food security [1,2]. Poultry farmers and stakeholders rely heavily on pharmaceutical products, primarily vaccines, vitamins, minerals, and antibiotics, to meet the increasing demand. Globally, poultry farmers use a large quantity of antibiotics not only for therapeutic purposes but also as a preventive measure against diseases [3-5]. Antibiotics have been utilized to enhance the efficiency of poultry production, enabling the production of high-quality poultry products at a reasonable cost and making them available to consumers. Many of these antimicrobials used in Bangladesh are essential in human medicine [6]. However, the indiscriminate use of these essential antimicrobials in animal production has been associated with the accelerated development of antimicrobial resistance (AMR) in pathogens, as well as in commensal organisms [4,7].

AMR has become one of the most important health problems of the 21st century, and it is of public importance [8,9]. When AMR is developed in bacteria, their susceptibility to antimicrobials is lost [10]. These resistant bacteria then multiply and become the dominant population, and as such, can transfer (both horizontally and vertically) the genes responsible for their resistance to other bacteria [11]. Humans can become infected with antibiotic-resistant bacteria by consuming and handling contaminated poultry meat [12].

*Salmonella* spp. causes human illnesses worldwide [13]. It can survive on a diverse range of food types, including poultry meat, pork, and vegetables. Chicken products are a major reservoir of *Salmonella* spp. [14]. Therefore, *Salmonella* leads to foodborne diseases, resulting in significant economic losses and deaths [15]. The emergence of AMR among *Salmonella* strains in chicken products has raised concern about using antimicrobials in poultry feeding practices [16,17]. Consumption of poultry infected with salmonellosis is considered a risk factor for transmission of multidrug-resistant (MDR) *Salmonella* from poultry to humans [18,19]. Salmonellosis caused by antibiotic-resistant *Salmonella* leads to prolonged illness, longer hospital stays, higher treatment costs, and a two-fold increased risk of post-infection morbidity [20]. *Salmonella* strains are long-lasting and may be resistant to common antibiotics, such as ampicillin, tetracycline (*tet*A), and chloramphenicol, due to their ability to form biofilms on poultry house surfaces [21].

Many researchers in Bangladesh isolated *Salmonella* spp. from different poultry sources and performed antibiotic sensitivity tests [22–25]. However, the occurrence of MDR *Salmonella* spp. in commercial poultry in Bangladesh over time has yet to be investigated. Investigating *Salmonella* spp. over a period is crucial, as it can reveal the progression of antibiotic resistance in birds during rearing. Long-term surveillance can help identify resistance trends among bacteria, facilitating the development of effective interventions. Therefore, the present investigation was conducted to detect antibiotic-resistant *Salmonella* spp. in commercial layer birds through phenotypic and genetic characterization, as well as their antibiotic resistance patterns.

## Materials and Methods

### Ethical approval

The study protocol was approved by the Animal Welfare and Experimentation Ethics Committee of Bangladesh Agriculture University, Mymensingh (Approval No. AWEEC/BAU/2018(27).

### Pre-work survey and study design

**Figure 1.**
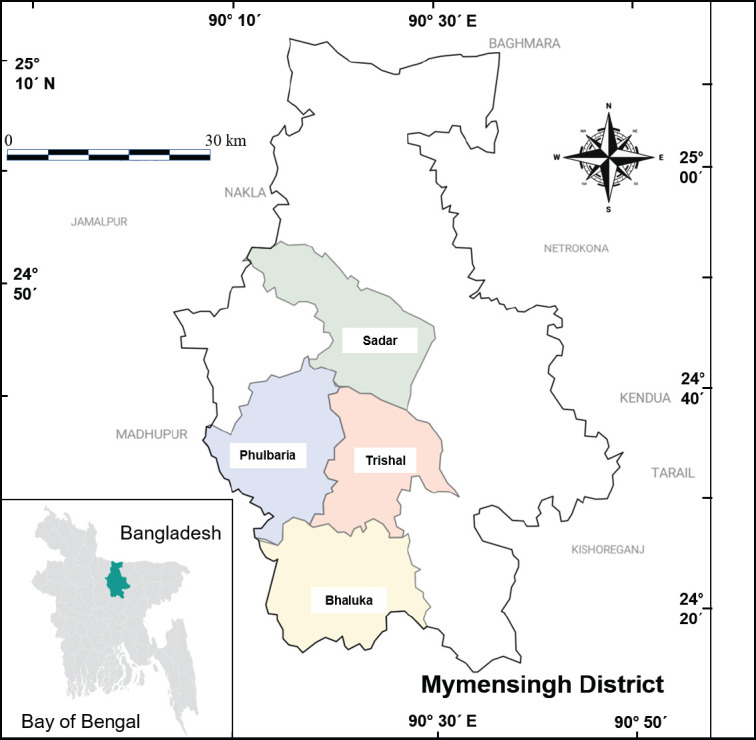
Sample collection sites (Bhaluka- 24° 22’N and 90° 22’E, Phulbaria- 24° 38’N and 90° 16’E, Trishal- 24° 32’N and 90° 20’E, and Sadar Upazila-24° 45’N and 90° 25’E).

Cloacal swab samples were collected from seven-layer farms across different areas of Mymensingh district (Bhaluka, Phulbaria, Trishal, and Sadar Upazila) (Fig. 1) and analyzed at the Department of Microbiology and Hygiene, Faculty of Veterinary Science, Bangladesh Agricultural University, Mymensingh, Bangladesh. Data was collected before initiating the experiment to assess the ‘antibiotics-using scenarios’ in the selected commercial layer farms. The data were collected through in-person interviews, during which the farm owners (from whom samples were intended to be collected) were asked predetermined questions. The survey aimed to collect information about the number and types of antibiotics used by the farmers during the study period. The study resumed with the meticulous collection of samples from egg-laying birds to isolate and confirm the presence of MDR *Salmonella* spp.

### Collection of samples and bacterial enrichment

A total of 105 samples were aseptically collected from the cloacal region of commercial layers in Eppendorf tubes with nutrient broth using sterile cotton swabs. Seven commercial layer farms were selected, with four located in Trishal and one farm representing each of the remaining locations. From each of the farms, 15 cloacal swabs were collected over a 4-month period of rearing. All samples were immediately placed on ice in a thermos flask after collection and transported to the laboratory. Then, the sample containing nutrient broth was placed in a bacteriological incubator and overnight incubated at 37°C.

### Isolation of Salmonella and genomic DNA extraction

A previously described procedure was followed for the isolation of *Salmonella* spp. [26]. Briefly, a small quantity of the bacteria-enriched broth was streaked on *Salmonella*-Shigella media and incubated overnight. DNA was extracted from the culture-positive isolates using the boiling method, as described previously [27].

### Molecular detection of Salmonella spp.

The *inv*A gene was targeted for amplification using a 25 µl reaction mixture comprised of 1µl of specific primers (Forward: 5’-ATC AGT ACC AGT CGT CTA TCT TGAT-3’ and Reverse: 5’-TCT GTT TAC CGG GCA TAC CAT-3’), 12.5 µl of 2× Master Mix (Promega, USA), 6.5 µl of nuclease-free water, and 4 µl of DNA template. The following thermal profile was inserted into the thermal cycler (Thermo Fisher Scientific, USA): Initial denaturation at 94°C for 5 min followed by 30 cycles of denaturation at 94°C for 30 sec, annealing at 52°C for 1 min, extension at 72°C for 45 sec, and final extension at 72°C for 5 min [28] ***.*** The amplified products were resolved on a 1.5% agarose gel, stained with ethidium bromide, and visualized under a UV-transilluminator (BIO-RAD, USA).

### Antibiotic sensitivity test using the disc diffusion method

The disc diffusion method was used to detect AMR patterns following the recommendations of the Clinical and Laboratory Standards Institute (CLSI) [29]. The antibiotic sensitivity patterns of *Salmonella* spp. isolates were evaluated using 19 antibiotics representing 7 different classes. The process began with the initial enrichment of the polymerase chain reaction (PCR)-positive isolates in nutrient broth, followed by spreading the enriched broth onto Mueller-Hinton agar after comparing it with the 0.5 McFarland standards. Discs of 19 antibiotics from seven classes- Aminoglycosides (Amikacin 30 µg, Gentamicin 10 µg, Streptomycin 10 µg, Neomycin 30 µg), *tet*A (*tet*A 30 µg, Doxycycline 30 µg), β-lactams [Cephalosporins (Cefixime 5 µg, Ceftriaxone 30 µg, Cefalexin 30 µg, Cefuroxime 30 µg) and Penicillin (Amoxicillin 10 µg, Ampicillin 10 µg)], Macrolides (Azithromycin 15 µg, Erythromycin 15 µg), Fluoroquinolones (Nalidixic acid 30 µg, Ciprofloxacin 5 µg, Levofloxacin 5 µg, Pefloxacin 5 µg), and Polymyxin (Colistin sulphate 10 µg) (Oxoid, UK) were inserted onto agar plates and incubated overnight at 37°C. Following the CLSI guidelines [29], the diffusion zones were measured.

### Detection of tetA and ampicillin-resistant genes in Salmonella spp. isolates

All phenotypically ampicillin- and *tet*A-resistant isolates were tested for the presence of resistance genes. To do so, the beta-lactamase(*bla*_TEM_) and *tet*A genes were targeted for amplification using a reaction mixture with the earlier composition and specific primer sets and the thermal profile (Table 1). A 1.5% agarose gel was prepared for electrophoresis, followed by staining and visualization under a UV transilluminator (BIO-RAD, USA).

**Table 1.** Thermal profiles for the detection of *tet*A and ampicillin-resistant genes in *Salmonella*.

Target gene	Thermal profiles						Size (bp)	References
	Initial denaturation	Denaturation	Annealing	Extension	Final extension	Cycles		
*tet*A	F: 5'-GGT TCA CTC GAA CGA CGT CA-3' R: 5'-CTG TCC GAC AAG TTG CAT GA-3'					34	577 bp	[30]
	95°C for 5 min	95°C for 1 min	54°C for 1 min	72°C for 1 min	72°C for 7 min			
*bla*TEM	5'-CAT TTC CGT GTC GCC CTT AT-3' 5'-TCC ATA GTT GCC TGA CTC CC-3'						793 bp	[31]
	95°C for 5 min	95°C for 1 min	56°C for 1 min	72°C for 1 min	72°C for 7 min			

### Statistical analysis

A chi-square (goodness-of-fit) test was conducted to evaluate whether the number of PCR-positive *Salmonella* isolates differed significantly across the seven commercial layer farms, each contributing an equal number of samples (*n* = 15). To assess statistically significant variations in resistance patterns among different farms and bird age groups, One-way Analysis of Variance (ANOVA) was performed. To determine the variation in inhibition zones across farms, the *F*-test was applied separately for each antibiotic. Statistically significant differences with *p*-values < 0.05 were considered. IBM SPSS Statistics for Windows, Version 29.0 (IBM Corp, NY, USA) was used for all statistical analyses.

## Results

### A pre-work survey suggests indiscriminate use of antibiotics

**Figure 2.**
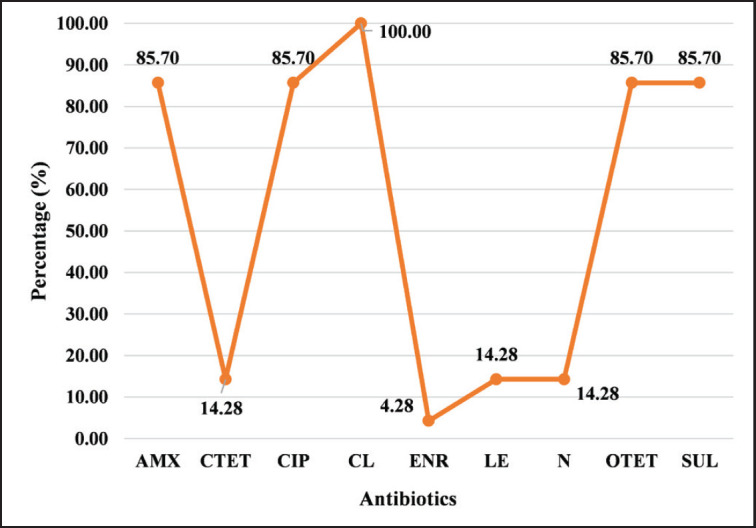
The overall rate of antimicrobials used randomly by farmers of selected layer farms. AMX, Amoxicillin; CTET, Chlortetracycline; CIP, Ciprofloxacin; CL, Colistin sulphate; ENR, Enrofloxacin; LE, Levofloxacin; N, Neomycin; OTET, Oxytetracycline; SUL, Sulfa drug.

Among the chosen layer farms, amoxicillin, ciprofloxacin, oxytetracycline, colistin sulphate, and sulfa drugs were predominantly used, which are considered crucial for human therapeutic use (Table 2). During the study period, 100% of the farmers used colistin sulfate, while 85.7% used a combination of amoxicillin, ciprofloxacin, oxytetracycline, and sulfa drugs, and 14.28% used chlortetracycline, levofloxacin, and neomycin. Enrofloxacin was used less frequently by the farm owners of the study area (4.28%) (Fig. 2). Overall, most farmers used a range of antibiotics during the study period, suggesting an indiscriminate use of essential antibiotics across the selected commercial layer farms.

**Table 2.** List of antimicrobials randomly used by the farmers of selected layer farms (color depicts the positive response. Separate colors indicate different antibiotics).

**Layer farm**	**Name of antibiotics**								
	AMX	CTET	CIP	CL	ENR	LE	N	OTET	SUL
Farm A									
Farm B									
Farm C									
Farm D									
Farm E									
Farm F									
Farm G									

AMX, Amoxicillin; CTET, Chlortetracycline; CIP, Ciprofloxacin; CL, Colistin sulphate; ENR, Enrofloxacin; LE, Levofloxacin; N, Neomycin; OTET, Oxytetracycline; SUL, Sulfa drug.

### Detection and confirmation of Salmonella spp.

**Figure 3.**
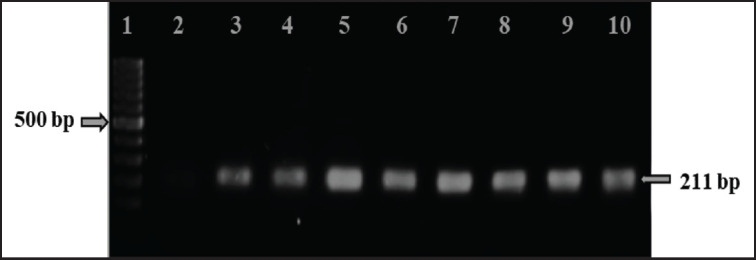
Amplification of the *inv*A (211 bp) gene from the Salmonella genus was carried out. Lane 1 was loaded with a 100 bp DNA ladder, Lane 2 with the negative control, Lane 3 with the positive control, and Lanes 4–10 with DNA templates of Salmonella spp.

The primary enrichment of bacteria in the nutrient broth was confirmed by turbidity. Following streaking, isolates produced round, black-centered colonies on SS agar media and were preliminarily selected as *Salmonella* spp. Among 105 samples, 34 isolates were suspected of being *Salmonella* spp. after culture. Of these culture-positive isolates, 20 were confirmed to carry the *inv*A gene by PCR, with bands observed at 211 bp after gel electrophoresis (Fig. 3). Overall, 19.05% of isolates were confirmed to be *Salmonella* spp. The chi-square test showed no significant difference in *Salmonella* distribution among farms (*χ*² = 3.834, df = 6, *p* = 0.70), indicating that the observed variation could be attributed to random chance (Table 3).

**Table 3.** Isolation of *Salmonella* spp. by culture and molecular methods from commercial layers.

Farm name	No. of samples	No. of culture-positive isolates	No. of PCR positive isolates	χ² value	*p*-value
A	105	3	1	3.834	*p* > 0.05*
B		6	5		
C		4	3		
D		6	4		
E		4	2		
F		6	3		
G		5	2		
Total		34	20 (19.05%)		

*A *p*-value greater than 0.05 (*p* > 0.05) was regarded as statistically insignificant.

### Salmonella isolates showed resistance to several classes of antibiotics

**Figure 4.**
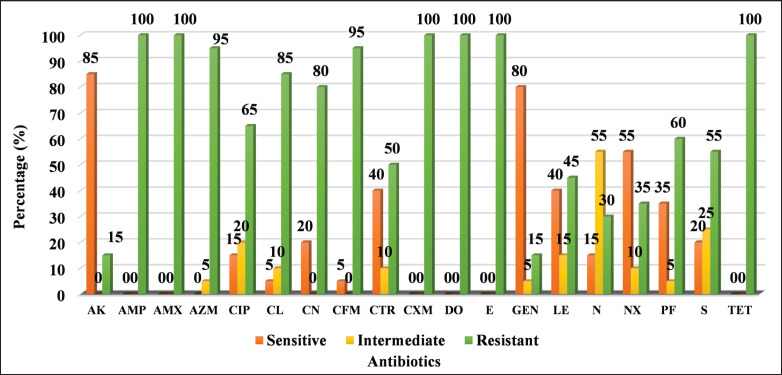
The overall antibiotic sensitivity/resistance patterns of Salmonella spp. isolated from commercial layers was determined. AK, Amikacin; AMP, Ampicillin; AMX, Amoxicillin; AZM, Azithromycin; CIP, Ciprofloxacin; CL, Colistin sulphate; CN, Cefalexin; CFM, Cefixime; CTR, Ceftriaxone; CXM, Cefuroxime; DO, Doxycycline; E, Erythromycin; GEN, Gentamicin; LE, Levofloxacin; N, Neomycin; NX, Nalidixic acid; PF, Pefloxacin; S, Streptomycin; TET, Tetracycline.

**Figure 5.**
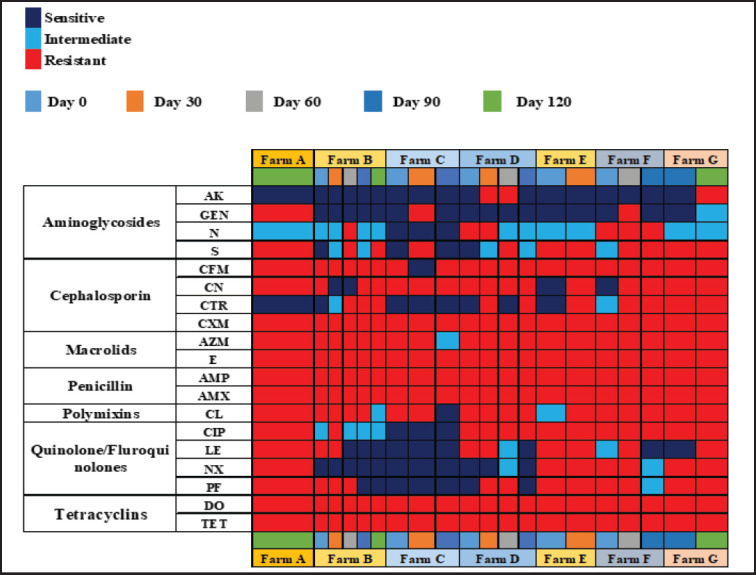
Antibiotic sensitivity/resistance patterns of Salmonella spp. isolated from commercial layers. AK, Amikacin; GEN, Gentamicin; N, Neomycin; S, Streptomycin; CFM, Cefixime; CN, Cefalexin; CTR, Ceftriaxone; CXM, Cefuroxime; AZM, Azithromycin; E, Erythromycin; AMP, Ampicillin; AMX, Amoxicillin; CL, Colistin sulphate; CIP, Ciprofloxacin; LE, Levofloxacin; NX, Nalidixic acid; PF, Pefloxacin; DO, Doxycycline; TET, Tetracycline. No quantitative scaling is applied.

As described previously, seven different classes of antibiotics were used to check the sensitivity/resistance profiles of *Salmonella* spp. The antibiogram results of the 20 PCR-positive *Salmonella* spp. isolates revealed diverse resistance patterns. 100% of the *Salmonella* spp. isolates showed resistance to ampicillin, amoxicillin, cefuroxime, doxycycline, erythromycin, and *tet*A (Fig. 4), while 95% of the isolates were resistant to azithromycin and cefixime. Approximately 80% were resistant to colistin sulphate and cefalexin, 65% and 60% to ciprofloxacin and pefloxacin, 55%, 50%, and 45% to streptomycin, ceftriaxone, and levofloxacin, respectively. Additionally, 30% and 35% were resistant to neomycin and nalidixic acid, respectively. Among the antibiotics tested, only amikacin and gentamicin demonstrated strong activity against *Salmonella* spp. isolated from commercial layers, with 85% of the isolates being sensitive to amikacin and 80% to gentamicin. Visualization of farm- and time-specific antibiotic resistance data in a heatmap revealed a range of sensitivity patterns (Fig. 5).

All the isolates were MDR; resistance extended from a minimum of four to a maximum of seven antibiotic classes. Notably, *Salmonella* spp. isolated on day 0 from Farms B, C, D, E, and F demonstrated resistance to 6, 5, 7, 6, and 7 antibiotic classes, respectively, indicating the presence of MDR *Salmonella* at an early age (Table 4). Statistical analysis revealed significant differences between several antibiotics. The greatest variation among farms was observed for ciprofloxacin (*F* = 27.63, *p* = 0.000001), followed by norfloxacin (*F* = 12.13, *p* = 0.000113), nalidixic acid (*F* = 4.68, *p* = 0.0095), and streptomycin (*F* = 4.46, *p* = 0.0115). These results indicate that resistance to these antibiotics significantly varies between farms. In contrast, no statistically significant differences were observed for certain antibiotics. Levofloxacin (*F* = 2.20, *p* = 0.110), gentamicin (*F* = 1.75, *p* = 0.187), ceftriaxone (*F* = 1.74, *p* = 0.189), amikacin (*F* = 1.52, *p* = 0.248), azithromycin (*F* = 0.92, *p* = 0.511), and cefixime (*F* = 0.92, *p* = 0.511) showed no significant variation in resistance among the farms. Statistical comparison using one-way ANOVA across bird age groups revealed no significant variation in AMR patterns. The uniform resistance profiles for several antibiotics likely contributed to the lack of statistical significance.

**Table 4.** antibiotic-resistant patterns according to the classes of antibiotics used against pcr-positive *salmonella* spp. isolates of each farm during the rearing period.

Farms	Age of the birds	Antibiotic sensitivity pattern (Group-wise)	No. of resistant antibiotic classes
A	Day 120	AMG-CEF-MAC-P-PM-Q-T	7
B	Day 0	CEF-MAC-P-PM-Q-T	6
	Day 30	CEF-MAC-P-PM-Q-T	6
	Day 60	AMG-CEF-MAC-P-PM-Q-T	7
	Day 90	CEF-MAC-P-PM-T	5
	Day 120	AMG-CEF-MAC-P-T	5
C	Day 0	CEF-MAC-P-PM-T	5
	Day 30	AMG-CEF-MAC-P-PM-T	6
	Day 90	CEF-MAC-P-T	4
D	Day 0	AMG-CEF-MAC-P-PM-Q-T	7
	Day 30	AMG-CEF-MAC-P-PM-Q-T	7
	Day 60	AMG-CEF-MAC-P-PM-Q-T	7
	Day 90	CEF-MAC-P-PM-Q-T	6
E	Day 0	AMG-CEF-MAC-P-Q-T	6
	Day 30	AMG-CEF-MAC-P-PM-Q-T	7
F	Day 0	AMG-CEF-MAC-P-PM-Q-T	7
	Day 60	AMG-CEF-MAC-P-PM-Q-T	7
	Day 90	AMG-CEF-MAC-P-PM-Q-T	7
G	Day 90	AMG-CEF-MAC-P-PM-Q-T	7
	Day 120	AMG-CEF-MAC-P-PM-Q-T	7

AMG-Aminoglycosides, CEF-Cephalosporins, MAC- Macrolides, P-Penicillin, PM-Polymyxins, Q-Quinolones/Fluoroquinolones, T-Tetracyclines.

### Detection of tetA and ampicillin-resistant genes in Salmonella spp. isolates

**Figure 6.**
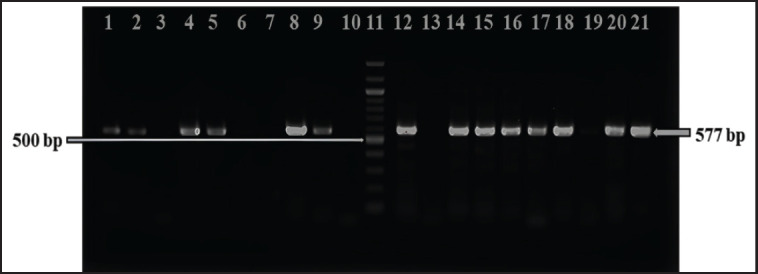
Amplification of the *tet*A (577 bp) gene in isolated Salmonella spp. was performed. Lane 11 was loaded with a 100 bp DNA ladder, Lane 12 with the positive control, Lane 13 with the negative control, and Lanes 1–10 and 14–21 with the amplified products of DNA samples of Salmonella spp.

**Figure 7.**
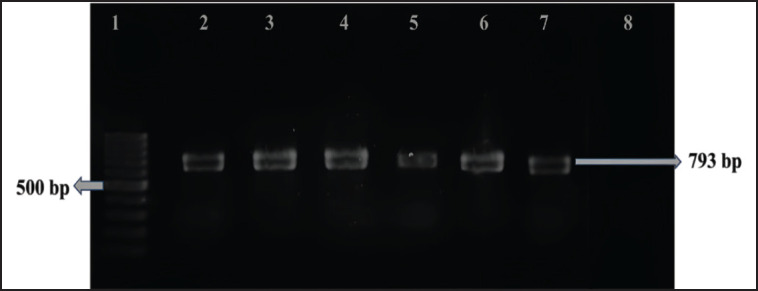
Amplification of the *bla*^TEM^ (793 bp) gene in isolated *Salmonella* was carried out. Lane 1 was loaded with a 100 bp DNA ladder, Lanes 2–6 with the amplified products of DNA samples of *Salmonella* spp., Lane 7 with the positive control, and Lane 8 with the negative control.

We examined all phenotypically *tet*A and ampicillin-resistant *Salmonella* spp. isolates to determine their genotypic resistance through PCR and gel electrophoresis by using specific primers. Under a UV-transilluminator, we observed positive bands at 577 and 793 bp for the *tet*A-resistant *tet*A gene and the ampicillin-resistant *bla*_TEM_ genes, respectively. All phenotypically *tet*A-resistant isolates were confirmed to carry *tet*A gene. In contrast, the ampicillin-resistant *bla*_TEM_ gene was confirmed in 13 of the 20 isolates (65%) (Figs. 6 and 7).

## Discussion

Antibiotics serve as the foundation for treating bacterial infections. However, this process can take a U-turn in the presence of antibiotic-resistant pathogens. *Salmonella* spp. are bacteria that have immense disease-producing potential for both humans and animals. The study also aimed to isolate antibiotic-resistant *Salmonella* spp. and examine their resistance patterns. The survey report showed the scenario of the most exploited antibiotics in poultry farming, as also reported by others [32]. Although colistin sulphate is classified as a reserve drug, it was used in 100% of the farms included in the study. In this study, 20/105 samples were confirmed to harbor *Salmonella* spp., corresponding to an isolation rate of 19.05%. This isolation rate was slightly higher than those documented in other studies, which reported isolation rates of 11.50%, 12.5%, and 12.5%, respectively [33–35]. The overall occurrence of *Salmonella* varied from farm to farm, and these differences may have been influenced by biosecurity and management factors, for example, the farm environment, hygiene, and the health status of the birds.

A total of 19 antibiotics were employed to test the isolates. These antimicrobials were classified into seven groups: quinolones, phenicols, β-lactams (including cephalosporins and penicillins), aminoglycosides, polymyxins, and *tet*A. The antibiogram of PCR-positive isolates revealed complete resistance (100%) to erythromycin, cefuroxime, doxycycline, amoxicillin, ampicillin, and *tet*A. High sensitivity was observed against amikacin (85%), gentamicin (80%), and nalidixic acid (55%). One prior study revealed that 100% of *Salmonella* isolates were sensitive to chloramphenicol and streptomycin, while 92 isolates (58%) were sensitive to nalidixic acid [36]. Like this study, previous research reported that 100% of the *Salmonella* spp. isolates were *tet*A resistant [37]. Resistance rates of streptomycin (64.5%) and nalidixic acid (39.5%) reported in another study were within a close range of this study [38]. Moreover, the results of this study align with another report, where all *Salmonella* spp. isolates from commercial layer flocks in the UK were sensitive to amikacin [39]. Sensitivity to ciprofloxacin and amikacin in 87.88% of *Salmonella* isolates was reported earlier [40].

Meanwhile, poultry-origin isolates were observed to be resistant to multiple classes of antibiotics, thereby signifying MDR. This concords with the findings from broiler farms, where 98% of the isolates were MDR [41]. It was observed that most isolates exhibited resistance to more than 10 antibiotics. A similar finding was reported in another study, where two *Salmonella* isolates were resistant to as many as 10 antibiotics, while the remaining isolates were mostly resistant to more than seven antibiotics [42]. This study showed that MDR *Salmonella* spp. is prevalent among the layer birds at an early age. Moreover, multiple classes of antibiotics were found to be ineffective against *Salmonella* spp. isolates. Some possible explanations for the presence of bacteria in young birds include vertical transmission from egg-laying hens, inadequate biosecurity measures at the hatchery and farm levels, and the consumption of contaminated feed and water. It is well-documented that *Salmonella* spp. can be transmitted vertically [43]. Additionally, poor biosecurity can facilitate the introduction of *Salmonella* spp. into hatcheries and farms where chicks are reared [18].

In this study, 100% and 65% of phenotypically *tet*A and ampicillin-resistant isolates contained *tet*A and *bla*_TEM_ genes, respectively. Consistent with the present study, a previous report also detected a high percentage of *Salmonella* spp. isolates carrying the* tet*A gene [44], but a different study reported a lower isolation rate (62%) of this gene [45]. However, it was reported that the *bla*_TEM_ gene was harbored by 82.9% of *Salmonella* spp. isolated from food animals, which is higher than that of this study [46]. The variations between this study and others may be due to differences in sampling areas, individual factors, nutrition, and health conditions. It is recommended that farm owners avoid the irrational use of antibiotics during the rearing period. Antibiotics should be used only when prescribed by a registered veterinarian. Biosecurity measures should be encouraged to reduce the bacterial burden on farms. Alternatives to antibiotics, such as probiotics, should be emphasized.

## Conclusion

Our study shows that *Salmonella* spp. is prevalent in commercial layer farms, and antibiotic resistance develops on these farms over time. According to the findings, layer birds can disseminate *Salmonella* spp. containing antibiotic-resistant genes in the farm environment. Given their increasing significance for public health, irrational use of antibiotics in layer farms for treatment and prevention should be discouraged. The adoption of strict biosecurity practices, along with the application of probiotics instead of antibiotics, has been recommended to address this issue.

## List of abbreviations

gm, gram; µl, microliter; °C, degree Celsius; min, minutes; spp., species; AMR, antimicrobial resistant; *bla*_TEM,_ β-lactamase; bp, base pair; CL, commercial layer; CLSI, clinical & laboratory standard institute; *inv*A, invasion A; MDR, multidrug-resistant; PCR, polymerase chain reaction; SS, Salmonella-Shigella; *tet*A, tetracycline A; UV, Ultra-violet.
